# Integrating Different Lines of Evidence to Establish a Novel Ascomycete Genus and Family (*Anastomitrabeculia*, *Anastomitrabeculiaceae*) in *Pleosporales*

**DOI:** 10.3390/jof7020094

**Published:** 2021-01-28

**Authors:** Chitrabhanu S. Bhunjun, Chayanard Phukhamsakda, Rajesh Jeewon, Itthayakorn Promputtha, Kevin D. Hyde

**Affiliations:** 1 Center of Excellence in Fungal Research, Mae Fah Luang University, Chiang Rai 57100, Thailand; avnishbhunjun@gmail.com (C.S.B.); chayanard91@gmail.com (C.P.); 2 School of Science, Mae Fah Luang University, Chiang Rai 57100, Thailand; 3 Engineering Research Center of Chinese Ministry of Education for Edible and Medicinal Fungi, Jilin Agricultural University, Changchun 130118, China; 4 Department of Health Sciences, Faculty of Medicine and Health Sciences, University of Mauritius, Reduit, Mauritius; r.jeewon@uom.ac.mu; 5 Department of Biology, Faculty of Science, Chiang Mai University, Chiang Mai 50200, Thailand; itthayakorn.p@cmu.ac.th

**Keywords:** BEAST, *Dothideomycetes*, *Pleosporales*, *Poaceae*, taxonomy, three new taxa, trabeculate pseudoparaphyses

## Abstract

A novel genus, *Anastomitrabeculia,* is introduced herein for a distinct species, *Anastomitrabeculia didymospora*, collected as a saprobe on dead bamboo culms from a freshwater stream in Thailand. *Anastomitrabeculia* is distinct in its trabeculate pseudoparaphyses and ascospores with longitudinally striate wall ornamentation. A new family, *Anastomitrabeculiaceae,* is introduced to accommodate *Anastomitrabeculia*. *Anastomitrabeculiaceae* forms an independent lineage basal to *Halojulellaceae* in *Pleosporales* and it is closely related to *Neohendersoniaceae* based on phylogenetic analyses of a combined *LSU, SSU* and *TEF1α* dataset. In addition, divergence time estimates provide further support for the establishment of *Anastomitrabeculiaceae.* The family diverged around 84 million years ago (MYA) during the Cretaceous period, which supports the establishment of the new family. The crown and stem age of *Anastomitrabeculiaceae* was also compared to morphologically similar pleosporalean families.

## 1. Introduction

*Pleosporales* is the largest order within *Dothideomycetes* (*Ascomycota*) [1]. The taxonomic and phylogenetic relationships of families and genera within this order are well documented [1,2,3,4,5,6,7]. *Pleosporales* comprises two suborders, *Massarineae* and *Pleosporineae* [1]. *Pleosporineae* includes economically important plant pathogens and *Massarineae* includes mainly saprobes from terrestrial or aquatic environments [1,3]. Zhang et al. [1] revised 174 genera and accepted 26 families in *Pleosporales*. The suborder *Massarineae* was resurrected to accommodate five families, the *Lentitheciaceae*, *Massarinaceae*, *Montagnulaceae* (*Didymosphaeriaceae*), *Morosphaeriaceae* and *Trematosphaeriaceae* [1]. Hyde et al. [2] correlated morphology with phylogenetic evidence and accepted 41 families in this order. Tanaka et al. [3] introduced two new families, *Parabambusicolaceae* and *Sulcatisporaceae*, accepting 12 families in *Massarineae*. The family *Longipedicellataceae* was introduced, and the divergence time in *Pleosporales* was estimated with emphasis on *Massarineae* [4]. The crown age of *Pleosporales* was dated to 211 MYA and *Massarineae* was dated to 130 MYA [4]. Species boundaries in *Cucurbitariaceae* were revised [5] and the family, *Lentimurisporaceae*, was introduced in *Pleosporales* [6].

Species in this order are abundant and occur in terrestrial, marine and freshwater habitats [7,8,9]. The species can be epiphytes, endophytes or parasites of living leaves or stems, hyperparasites on fungi or insects, lichenized, or saprobes of dead plant stems, leaves or bark [7,8,9]. Currently, about 400 genera in 64 families are known in *Pleosporales* [1,2,7,10,11,12,13], with numerous coelomycetous and hyphomycetous taxa as their asexual morphs [1,13,14,15].

Several pleosporalean taxa are pathogens associated with a broad range of hosts including bamboo. Bamboo (*Poaceae*) comprises over 115 genera with around 1500 species [16,17,18], can be found in diverse climates [17], and are widely distributed in various forest types in Thailand [18,19]. It has been estimated that around 1100 fungal species belonging to over 200 genera have been described or recorded worldwide on bamboo and most of these bamboo-associated fungi are ascomycetes [20,21].

Divergence time estimates using molecular clock methodologies have been widely used in fungal taxonomy [4,11,22,23,24,25,26,27]. Several studies have applied molecular dating to provide additional evidence for higher taxa ranking in *Pleosporales* [4,6,7,11]. In this study, we introduce a novel bambusicolous species, *Anastomitrabeculia didymospora* within *Anastomitrabeculia*, which is accommodated in a new family, *Anastomitrabeculiaceae*, based on morphology, multi-loci phylogeny and divergence times estimates.

## 2. Materials and Methods

### 2.1. Sample Collection, Isolation and Identification

Dead bamboo culms were collected from a freshwater stream from Krabi province, Thailand, in 2015. The samples were incubated in plastic boxes with sterile and moist tissue at 25–30 °C for 3 days. Pure fungal colonies were obtained using single-spore isolation [28]. Germinating spores were transferred aseptically to potato dextrose agar (PDA) and malt extract agar (MEA) (Difco™). The cultures were incubated at 25 °C with frequent observations. Fungal characters were observed using a stereo microscope (Zeiss SteREO Discovery v. 8) fitted with an Axio Cam ERc5S and a Leica DM2500 compound microscope attached with a Leica MC190 HD camera. All microscopic measurements were carried out using Tarosoft (R) Image Frame Work program and the images were processed with Adobe Photoshop CS6 version 13.0 software (Adobe Systems, San Jose, CA, USA). The type specimens were deposited in the Mae Fah Luang University (MFLU) Herbarium, Chiang Rai, Thailand, and pure cultures were deposited at the Mae Fah Luang University Culture Collection (MFLUCC). The new taxon was linked with Facesoffungi numbers (FoF) [29] and Index Fungorum (Index Fungorum 2020, http://www.indexfungorum.org/, accessed on 2 December 2020) and established based on guidelines recommended by Jeewon and Hyde [30].

### 2.2. DNA Extraction, PCR Amplification and DNA Sequencing

DNA extraction, PCR amplification, DNA sequencing and phylogenetic analysis were carried out as detailed in Dissanayake et al. [31]. Total genomic DNA was extracted from fresh mycelium with a Biospin Fungus Genomic DNA Extraction Kit (BioFlux^®^) (Hangzhou, P.R. China) following the manufacturer’s protocol. The nuclear ribosomal large subunit 28S rRNA gene (*LSU*) [32], the nuclear ribosomal small subunit 18S rRNA gene (*SSU*) [33] and the translation elongation factor 1-alpha gene (*TEF1α*) [34] were amplified using primers (*LSU*: LROR/LR5, *SSU*: NS1/NS4 and *TEF1α*: 983F/2218R). Polymerase chain reaction (PCR) was performed using PCR mixtures containing 5–10 ng DNA, 1X PCR buffer, 0.8 units Taq polymerase, 0.3 μM of each primer, 0.2 mM dNTP and 1.5 mM MgCl_2_. PCR conditions were set at an initial denaturation for 3 min at 94 °C, followed by 40 cycles of 45 s of denaturation at 94 °C, annealing for 50 s at 56 °C for *LSU*, *SSU* and 52 °C for *TEF1α* and extension for 1 min at 72 °C, with a final extension of 10 min at 72 °C. All the PCR products were visualised on 1% Agarose gels with added 6 μL of 4S green dyes, per each 100 mL. Successful PCR products were purified and sequencing was performed by Shanghai Sangon Biological Engineering Technology & Services Co. (Shanghai, P.R. China). All sequences generated in this study were submitted to GenBank (Table 1) and the ITS region of *Anastomitrabeculia didymospora* was deposited with the accession number MW413900 (MFLUCC 16-0412) and MW413897 (MFLUCC 16-0417).

### 2.3. Phylogenetic Analysis

The sequence data were assembled using BioEdit v. 7.2.5 [35] and subjected to a BLAST search (https://blast.ncbi.nlm.nih.gov/Blast.cgi) to find the closest matches with taxa in *Pleosporales*. Reference sequence data of this order and some representatives of other orders of *Dothideomycetes* were downloaded from previously published studies [1,6,36,37,38,39]. The sequences were automatically aligned using default settings in MAFFT v. 7 (http://mafft.cbrc.jp/alignment/server/) [40]. A combined dataset of three gene regions (*LSU*, *SSU* and *TEF1α*) was prepared and manually adjusted using BioEdit and AliView [41]. Phylogenetic analyses of the combined dataset were performed using maximum likelihood, maximum parsimony and Bayesian inference method. Maximum likelihood analyses (ML), including 1000 bootstrap pseudoreplicates, were performed at the CIPRES web portal [42] using RAxML v. 8.2.12 [43]. Maximum parsimony analysis was conducted using PAUP v.4.0b 10 [44] with the heuristic search option and number of replications 1000 each. The Tree Length (TL), Consistency Indices (CI), Retention Indices (RI), Rescaled Consistency Indices (RC) and Homoplasy Index (HI) were documented.

The best model for different genes partition was determined in JModelTest version 2.1.10 [45] for posterior probability (PP). The general time reversible (GTR) model with a discrete gamma distribution plus invariant site (GTR+I+G) substitution model was used for the combined dataset. Posterior probabilities [46] were estimated by Markov Chain Monte Carlo sampling (MCMC) in MrBayes v. 3.2.6 [47]. Four simultaneous Markov chains were run for 10 million generations and trees were sampled every 1000th generation, thus resulting in 10,000 trees. The suitable burn-in phase was determined by inspecting traces in Tracer version 1.7 [48]. The first 10% of generated trees representing the burn-in phase of the analyses were discarded, while the remaining trees were used to calculate posterior probabilities (PP) in the majority rule consensus tree. The phylograms were visualized with FigTree v1.4.0 program [49] and edited using Adobe Illustrator CS6 v15.0 (Adobe Systems, USA).

### 2.4. Fossil Calibration and Divergence Time Estimates

Divergence times were estimated with BEAST 2.6.2 [50] based on the methodology described in Phukhamsakda et al. [4]. The aligned sequence dataset (*LSU*, *SSU* and *TEF1α*) used for the phylogenetic analyses were loaded into BEAUTI 2.6.2 to prepare the XML file. Nucleotide substitution models were determined using JModelTest version 2.1.10. The GTR+I+G nucleotide substitution model was applied to *LSU* and *TEF1α* partitions. The symmetrical (SYM) model with a discrete gamma distribution plus invariant site (SYM+I+G) substitution model was applied to the SSU partition. The data partitions were set with unlinked substitution, linked clock model and linked tree. An uncorrelated relaxed clock model with lognormal distribution was used. The Yule speciation process, which assumes a constant rate of speciation divergence, was used as the tree prior [51]. The analysis was performed in BEAST 2.6.2 for 100 million generations, sampling every 1000 generations. The effective sample size (ESS) was analysed with Tracer version 1.7 to check that the values were greater than 200, as recommended by Drummond et al. [52]. The first 20% trees were discarded as the burn-in phase and the remaining trees were combined in LogCombiner 2.6.2. The maximum clade credibility was calculated in TreeAnnotator v 2.6.2. The phylograms were visualized with FigTree v.1.4.0 program.

To estimate the divergence time for Anastomitrabeculiaceae, the fossil *Metacapnodium succinum* (*Metacapnodiaceae*) was used to set the crown age of *Capnodiales* using a normal distribution, mean of 100 MYA, SD of 150 MYA, giving 95% credibility interval of 346 MYA [4,23,53,54]. The fossil *Margaretbarromyces dictyosporus* was used to calibrate the crown age of *Aigialus* (*Aigialaceae*) using a gamma distribution, with an offset of 35 MYA, a shape of 1.0, scale of 25, providing 95% credibility interval of 110 MYA [4,55,56,57]. The split between *Arthoniomycetes* (outgroup) and *Dothideomycetes* was used as the secondary calibration using a normal distribution, mean of 300 MYA, SD of 50 MYA, giving 95% credibility interval of 382 MYA [22,36,53,54].

## 3. Results

### 3.1. Phylogenetic Analyses

**Figure 1:**
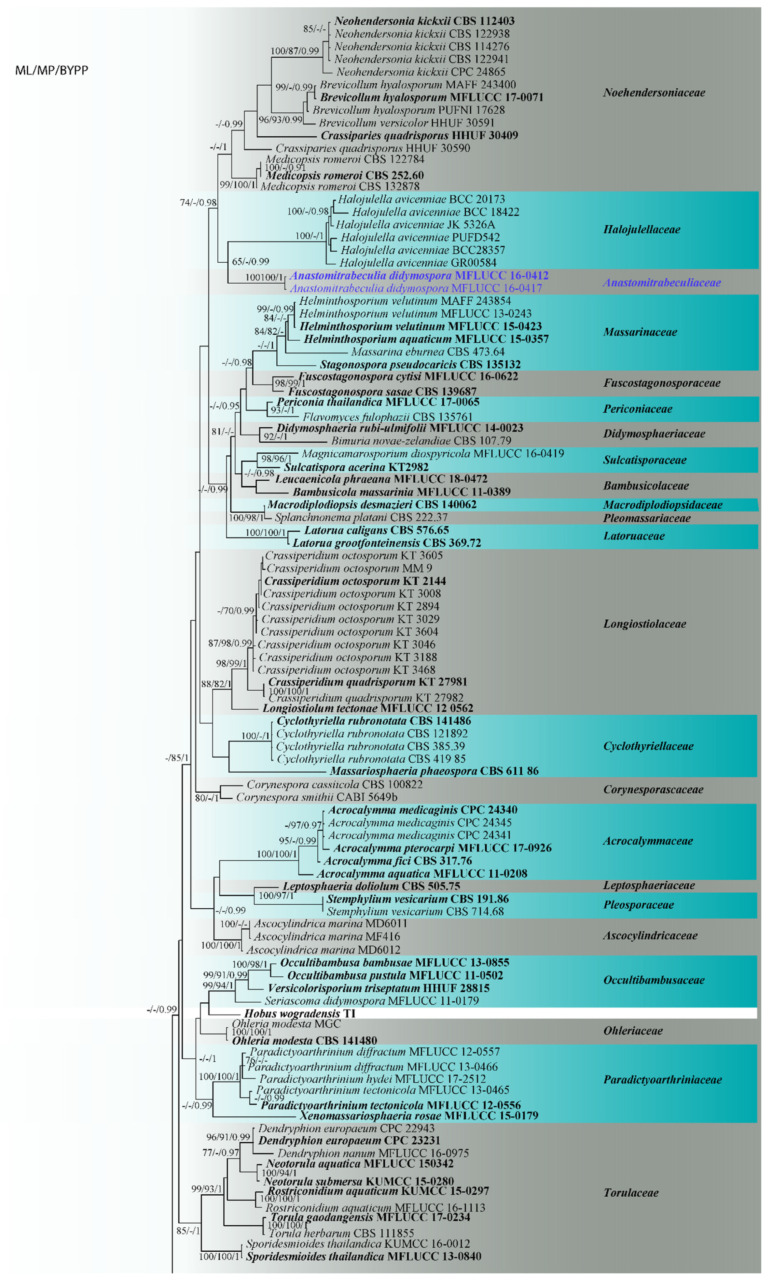

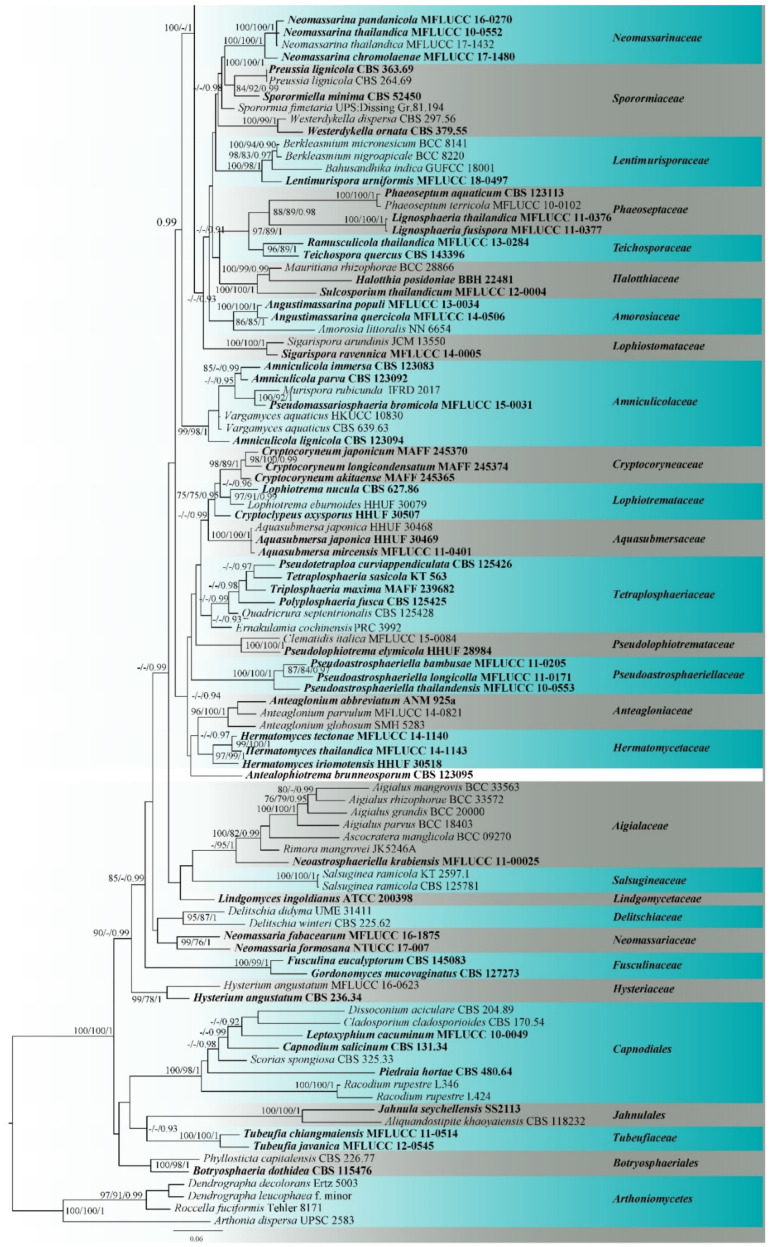
The best scoring RAxML tree based on a combined *LSU*, *SSU* and *TEF1α* dataset. RAxML bootstrap support and maximum parsimony values ≥60% (BT), as well as Bayesian posterior probabilities ≥0.90 (BYPP) are shown, respectively, near the nodes. The ex-type strains are in bold and the scale bar indicates 0.06 changes per site. The tree is rooted with species of *Arthoniomycetes* and the new taxon is indicated in blue.

The combined gene alignment comprised 196 strains and 2800 characters (*LSU*: 860 characters, *SSU*: 1039 characters and *TEF1α*: 901 characters). Among the 2800 characters, there were 1492 conserved sites (53%), 364 variable sites (13%) and 944 parsimony informative sites (34%). The parsimony analysis of the data matrix yielded one most parsimonious tree out of 1000 (CI = 0.265, RI = 0.659, RC = 0.175, HI = 0.735, Tree Length = 7606). Based on BLAST search in the NCBI GenBank of the *LSU* gene, the newly generated taxon MFLUCC 16-0412 and MFLUCC 16-0417 show 95% similarity to *Crassiperidium quadrisporum* (KT 27981 and KT 27982). The topology of the phylogenetic tree based on the *LSU* gene was generally congruent with the overall topology of the tree based on the combined dataset. Phylogenetic trees generated from maximum likelihood, maximum parsimony and Bayesian analysis of the combined dataset resulted in similar topologies with some exception. The position of *Cyclothyriellaceae* and *Longiostiolaceae* differed between the three methods. The best scoring RAxML tree had a final likelihood value of −40,523.297855 (Figure 1). The new taxon formed an independent lineage basal to the *Halojulellaceae* with strong Bayesian inference support and moderate support from maximum likelihood (0.99 PP/65% MLBT). A new genus *Anastomitrabeculia* is therefore introduced within *Anastomitrabeculiaceae* to accommodate the new species.

### 3.2. Fossil Calibration and Divergence Time Estimates

**Figure 2:**
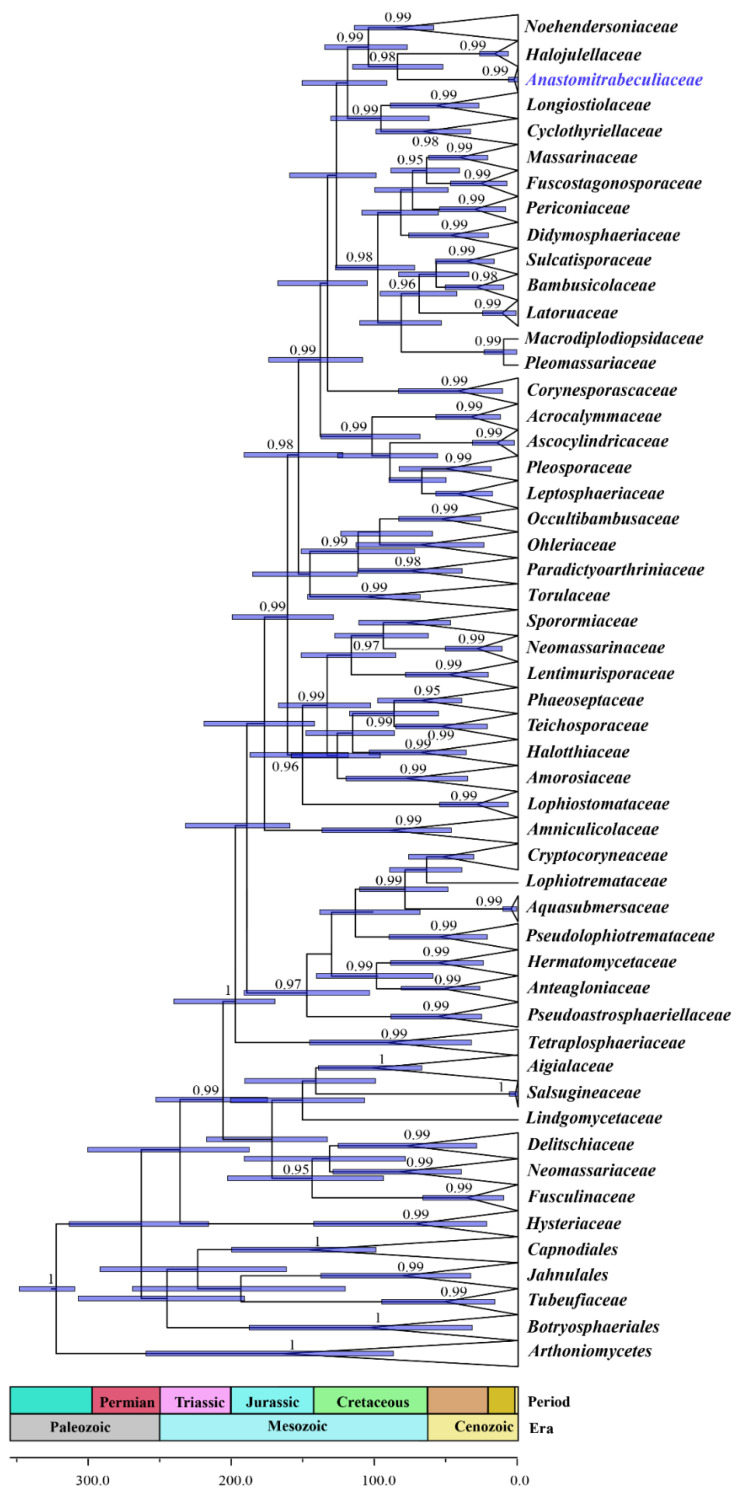
Maximum clade credibility (MCC) tree of families in *Dothideomycetes* using BEAST. Numbers at nodes indicate posterior probabilities (PP) for node support. Bars correspond to the 95% highest posterior density (HPD) intervals. Posterior probabilities greater than 0.95 are given near the nodes. The new taxon is indicated in blue. Geological time scales are given at the base together with scale in million years ago (MYA) [58].

The topology of the maximum clade credibility (MCC) tree (Figure 2) was congruent with the tree obtained from the Bayesian inference analysis and the maximum likelihood analysis. The divergence times of the dating analysis are listed in Table 2. The crown age of *Dothideomycetes* is estimated at 263 MYA during the Permian period based on the MCC tree. The split of *Arthoniomycetes* and *Dothideomycetes* occurred around 323 MYA during the Carboniferous period. The crown age of *Pleosporales* is estimated at 206 MYA, and *Hysteriales* diverged from *Pleosporales* approximately 236 MYA during the Triassic period. The crown age of *Anastomitrabeculiaceae* is estimated at around 2.6 MYA, and it diverged from *Halojulellaceae* at around 84 (52–116) MYA. *Anastomitrabeculiaceae* formed an independent lineage with close relationship to *Halojulellaceae* with strong posterior probability in the MCC tree (0.99 BYPP). The divergence time of *Anastomitrabeculiaceae* was compared to Pleosporalean families with trabeculate pseudoparaphyses, cylindrical asci and ascospores with a sheath (Table 3). The divergence time of *Anastomitrabeculiaceae* was also compared to *Didymosphaeriaceae* as they are morphologically similar by having trabeculate pseudoparaphyses and cylindrical asci.

### 3.3. Taxonomy

*Anastomitrabeculiaceae* Bhunjun, Phukhams and K.D. Hyde, *fam*. *nov*.

Index Fungorum number: IF556817, Facesoffungi number: FoF 09521.

Etymology: Referring to the name of the type genus.

Saprobic on dead bamboo culms submerged in freshwater. **Sexual morph**: *Ascomata* immersed under a clypeus to semi-immersed, gregarious, uniloculate, globose to subglobose, carbonaceous, black. *Ostiole* central, apex well developed. *Peridium* multi-layered, sub-carbonaceous or coriaceous, with dark brown to hyaline cells arranged in a *textura angularis*. *Hamathecium* composed of numerous, filamentous, trabeculate pseudoparaphyses, septate, anastomosing between the asci and at the apex. *Asci* bitunicate, fissitunicate, broad cylindrical to cylindrical-clavate, bulbous pedicel, with an ocular chamber. *Ascospores* biseriate, broadly fusiform, septate, smooth-walled, with wall ornamentation, surrounded by mucilaginous sheath.

Note: *Anastomitrabeculiaceae* is introduced to include *Anastomitrabeculia*, which is reported as a saprobe on bamboo culms. *Anastomitrabeculiaceae* is characterised by semi-immersed, coriaceous or carbonaceous ascomata with septate, trabeculate pseudoparaphyses and hyaline ascospores with longitudinally striate wall ornamentation, surrounded by mucilaginous sheath. *Anastomitrabeculiaceae* formed a well-supported independent lineage closely related to *Halojulellaceae*, but *Halojulellaceae* differs by its cellular pseudoparaphyses and golden-brown ascospores.

Type genus: *Anastomitrabeculia* Bhunjun, Phukhams and K.D. Hyde.

*Anastomitrabeculia* Bhunjun, Phukhams. and K.D. Hyde, *gen*. *nov*.
Index Fungorum number: IF556560, Facesoffungi number: FoF 09522.
Etymology: Referring to the trabeculate pseudoparaphyses anastomosing between the asci and at the apex.

Colonies on natural substrate umbonate at the centre, circular, black shiny dots are visible on the host surface. *Ascomata* on surface of the host, immersed under a clypeus, gregarious, uniloculate, subglobose, carbonaceous. *Ostiole* orange pigment near ostiole. *Peridium* comprising multilayers of brown to hyaline cells of *textura angularis*, inner layers composed of thin, hyaline cells. *Asci* 8–spored, bitunicate, fissitunicate, broad cylindrical to cylindrical-clavate, with a bulbous pedicellate, rounded at the apex, with an ocular chamber. *Ascospores* biseriate, broadly fusiform, tapering towards the ends, hyaline, with guttules in each cell, constricted at the septa, with longitudinally striate wall ornamentation, surrounded by mucilaginous sheath.

Note: *Anastomitrabeculia* is established as a monotypic genus. It is characterised by the presence of carbonaceous ascomata, with orange pigment near ostiole and ascospores with longitudinally striate wall ornamentation. *Anastomitrabeculia* is morphologically similar to members of *Pleosporales* in having perithecioid ascomata, bitunicate asci and hyaline ascospores.

Type species: *Anastomitrabeculia didymospora* Bhunjun, Phukhams and K.D. Hyde.

**Figure 3:**
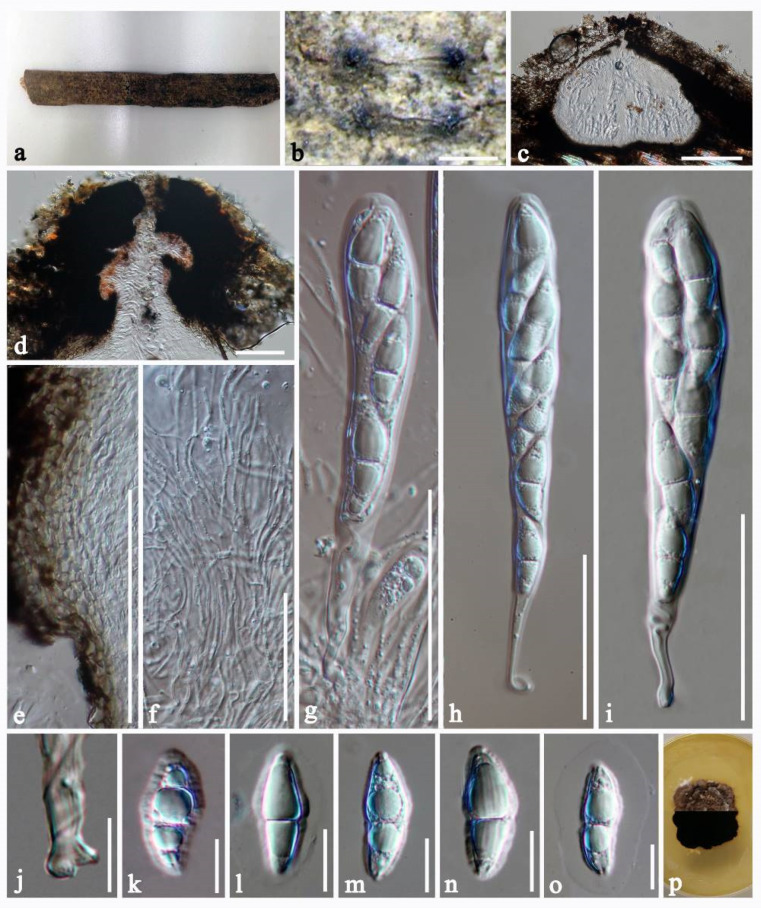
*Anastomitrabeculia didymospora* (MFLU 20-0694, **holotype**). (**a**) Ascomata on bamboo. (**b**) Close-up of ascomata. (**c**) Vertical section of ascoma. (**d**) Ostiolar canal. (**e**) Peridium layer. (**f**) Trabeculate pseudoparaphyses. (**g**–**i**) Asci. (**j**) Pedicel. (**k**–**o**) Ascospores showing mucilaginous sheath. (**p**) Culture characteristics on PDA from above and below (9 cm diameter petri dish). Scale bar: (**b**) = 500 µm, (**c**) = 200 µm, (**d**–**i**) = 50 µm, (**j**–**o**) = 10 µm.

Anastomitrabeculia didymospora Bhunjun, Phukhams and K.D. Hyde, sp. nov.
Index Fungorum number: IF556559; Facesoffungi number: FoF 09523 Figure 3.
Etymology: Referring to the didymosporous ascospores.
Holotype–MFLU 20-0694.

Saprobic on dead bamboo culms submerged in freshwater. **Sexual morph**: *Ascomata* 430–460 μm high, 435–575 μm diam., immersed under a clypeus to semi-immersed, gregarious, uniloculate, globose to subglobose, carbonaceous, rough, black, ostiolate. *Ostiole* 160 μm high, 270 μm diam., central, apex well developed, papillate, with pore-like opening, with periphyses filling the ostiolar canal, dark brown to black, orange pigment near ostiole. *Peridium* 6–18 μm wide, comprising 3–5 layers of brown to hyaline cells of *textura angularis*, inner layers composed of thin, hyaline cells. *Hamathecium* of dense, long, 0.8–1.25 µm wide ($\bar{x}$ = 1 μm, *n* = 50), filiform, filamentous, trabeculate pseudoparaphyses, septate, branched, embedded in a gelatinous matrix, anastomosing between the asci and at the apex. *Asci* 125–160 × 15–20 μm ($\bar{x}$ = 145 × 17 μm, *n* = 20), 8–spored, bitunicate, fissitunicate, broad cylindrical to cylindrical-clavate, with bulbous pedicellate, rounded at the apex, with an ocular chamber. *Ascospores* 18–28 × 7–10 μm ($\bar{x}$ = 22.5× 9 μm, *n* = 20), biseriate, broadly fusiform, tapering towards the ends, hyaline, 1-septate at the centre, constricted at the septum, cell above septate enlarged, straight, smooth-walled, with longitudinally striate wall ornamentation, surrounded by mucilaginous sheath. **Asexual morph:** Undetermined.

Culture characters: Ascospores germinating on MEA and PDA within 24 h with germ tubes developing from basal cells. Colonies on MEA and PDA umbonate at the centre, circular, friable, reaching 20 mm diameter after four weeks of incubation at 25 °C. Culture on MEA with white aerial mycelium, dark brown at the centre and paler towards the edge from above and below. Culture on PDA dark brown from above and below.

Material examined: THAILAND, Krabi province (8.1° N, 98.9° E), on dead bamboo culms, 15 December 2015, C. Phukhamsakda, KR001 (MFLU 20-0694, **holotype**), ibid, 18 December 2015 (MFLU 20-0695, **paratype**); ex-type living culture MFLUCC 16-0412; ex-paratype living culture, MFLUCC 16-0417.

## 4. Discussion

In this study, we introduce a new species, genus and family for a collection of *Pleosporales* found on bamboo. The introduction of new taxa, even at the family level, is not surprising, considering that about 93% of fungi remain unknown to science despite ca. 2000 species described every year [59,60]. Pleosporalean species can occur in terrestrial, marine and freshwater habitats [7,8,9]. Several studies have reported new pleosporalean taxa from freshwater or marine habitats or from bambusicolous hosts [1,3]. *Pleosporales* have unique characters such as perithecioid ascomata typically with a papilla and bitunicate, generally fissitunicate asci, bearing mostly septate ascospores of different colours and shapes, with or without a gelatinous sheath [7]. The morphology of *Anastomitrabeculiaceae* is similar to members of the *Pleosporales* based on the presence of pseudoparaphyses, perithecioid ascomata, bitunicate asci and hyaline ascospores. *Anastomitrabeculiaceae* is characterised by semi-immersed to superficial ascomata, trabeculate pseudoparaphyses, cylindrical asci and ascospores with longitudinally striate wall ornamentation, surrounded by mucilaginous sheath. The newly discovered species formed a well-supported independent lineage basal to the *Halojulellaceae* based on phylogenetic analyses of the combined dataset (0.99 PP/65% MLBT)*. Halojulellaceae* differs by its cellular pseudoparaphyses and golden brown ascospores [2]. The new taxon is also phylogenetically closely related to *Neohendersoniaceae*, which differs by its cellular pseudoparaphyses and smooth-walled ascospore [61]. A novel genus *Anastomitrabeculia* is therefore introduced to accommodate one new species, *Anastomitrabeculia didymospora.* A new family, *Anastomitrabeculiaceae*, is also introduced to accommodate this independent lineage.

Several pleosporalean families such as Aigialaceae, Amniculicolaceae, Anteagloniaceae, Astrosphaeriellaceae, Bambusicolaceae, Biatriosporaceae, Caryosporaceae, Cyclothyriellaceae, Delitschiaceae, Didymosphaeriaceae, Fuscostagonosporaceae, Lindgomycetaceae, Melanommataceae, Neomassariaceae, Pseudoastrosphaeriellaceae, Striatiguttulaceae and Tetraplosphaeriaceae share similar characters to Anastomitrabeculiaceae in having trabeculate pseudoparaphyses, cylindrical asci and ascospores with a sheath [7]. The nature of pseudoparaphyses is often overlooked, but they have taxonomic relevance at the genus and possibly family levels [7], but not at the ordinal level [62]. These families differ from Anastomitrabeculiaceae mainly by their ascospores, for example, Aigialaceae and Amniculicolaceae have brown and muriform ascospores [7]. Anteagloniaceae differs by having a peridium composed of dark brown cells of textura epidermoidea, cellular or trabeculate pseudoparaphyses and small, uniseriate ascospores [2]. Astrosphaeriellaceae differs by its brown, sub-fusiform to fusiform, obclavate to ellipsoidal, or limoniform ascospores [63] and Biatriosporaceae differs by its immersed ascomata and fusiform, dark brown ascospores [2]. Caryosporaceae differs by its broad-fusiform, ovoid or ellipsoid, brown ascospores [64]. Bambusicolaceae species have also been isolated from dead bamboo culms, but they differ from Anastomitrabeculiaceae by their cellular pseudoparaphyses and multi-seriate, smooth-walled ascospores [2]. Cyclothyriellaceae differs by its uniseriate, ellipsoid to fusiform, brown ascospores with several eusepta [65]. Fuscostagonosporaceae differs in having globose to subglobose ascomata, fissitunicate asci with long stipes and narrowly fusiform ascospores [66]. Anastomitrabeculiaceae shares several characters with Didymosphaeriaceae in having immersed ascomata formed under a clypeus, trabeculate pseudoparaphyses and cylindrical asci. Didymosphaeriaceae and Melanommataceae differ in having cellular or trabeculate pseudoparaphyses and brown, multi-septate, muriform ascospores [7]. Lindgomycetaceae differs by the presence of cellular or trabeculate pseudoparaphyses and brown, multi-septate ascospores with bipolar mucilaginous appendages [7]. Neomassariaceae differs by its immersed ascomata and ellipsoid ascospores. Pseudoastrosphaeriellaceae differs by its brown to reddish-brown ascospores with longitudinal ridges towards the ends and Striatiguttulaceae differs in having brown, ellipsoid ascospores with paler end cells. Tetraplosphaeriaceae differs by its immersed ascomata and slightly curved, pale brown ascospores [7].

Divergence time estimate has been widely used as supporting evidence to clarify taxonomic status of extant or novel families in fungal taxonomy [4,6,23,24,26,27,67]. In this study, the MCC tree was congruent with the topology of the trees generated from Bayesian inference analysis and maximum likelihood analyses. The divergence time estimates for the crown age of *Dothideomycetes* (263 MYA), the split of *Dothideomycetes* and *Arthoniomycetes* (323 MYA), the crown age of *Pleosporales* (206 MYA) and the divergence of *Hysteriales* from *Pleosporales* (236 MYA) are similar to previous studies [4,7,11]. Hyde et al. [27] recommended that the divergence times of families should be between 50 and 150 MYA. The stem age is usually preferred to the crown age in taxa ranking as it is not affected by the sample size of the clade [27]. Based on the MCC tree, *Anastomitrabeculiaceae* and *Halojulellaceae* share the stem age of 84 MYA which supports the establishment of *Anastomitrabeculiaceae*.

The divergence time of *Anastomitrabeculiaceae* was also compared to Pleosporalean families with trabeculate pseudoparaphyses, cylindrical asci and ascospores with a sheath (Table 3). *Cyclothyriellaceae* has an estimated crown age of 66 MYA and it diverged at 95 MYA. *Fuscostagonosporaceae* has a crown age of approximately 26 MYA and it diverged around 63 MYA. *Bambusicolaceae*, which was also isolated from dead bamboo culms, has a crown age of 29 MYA and a stem age of about 57 MYA. The stem age of *Anastomitrabeculiaceae* lies within the range of divergence times of those with similar morphology, but the crown age of *Anastomitrabeculiaceae* (2.6 MYA) is much earlier compared to these families. *Bambusicolaceae* was introduced by Hyde at al. [2] to include three bambusicolous taxa, and it currently has 15 species [7]. *Fuscostagonosporaceae* was introduced by Hyde at al. [66] to accommodate one bambusicolous taxon and it currently has four species [7]. Ariyawansa et al. [64] introduced the pleosporalean family, *Caryosporaceae*, which is morphologically similar to *Astrosphaeriellaceae* and *Trematosphaeriaceae* [7]. Based on Liu et al. [11], the stem age of *Caryosporaceae* (85 MYA) is similar to *Trematosphaeriaceae* (88 MYA) compared to *Astrosphaeriellaceae* (113 MYA), but the crown age of *Caryosporaceae* (2 MYA) is much earlier compared to *Astrosphaeriellaceae* (55 MYA) and *Trematosphaeriaceae* (65 MYA). *Astrosphaeriellaceae* currently has 111 species, and *Trematosphaeriaceae* has 103 species, whereas *Caryosporaceae* has ten species [7]. Compared to their morphologically similar families, the early crown of *Anastomitrabeculiaceae* and *Caryosporaceae* could be due to their smaller sample size. Therefore, further collections are needed for an accurate estimation of the crown age as it is affected by the sample size of the clade [27]. This could also be due to rapid speciation of pleosporalean fungal species given their high adaptation capabilities.

The estimated crown age of *Pleosporales* (206 MYA) lies within the early Triassic period. The origin of monocotyledons is estimated within the late Cretaceous period (around 145 MYA) [68]. This period is associated with the diversification of pleosporalean families, which continued during the early Cretaceous period when there was a major diversification and radiation of angiosperms, which favoured further diversification of Pleosporalean families to adapt to various hosts [69].

Hosts and their symbionts can speciate in parallel, which relates to a high level of congruence between the phylogeny of the hosts and their symbionts [70,71]. Therefore, studies focusing on divergence time is important for a better understanding of host–pathogen interaction as well as co-evolutionary interactions [72]. This study uses a polyphasic approach based on morphology, multi-locus phylogenetic analyses and divergence time estimates. By implementing a polyphasic approach, we provide strong evidence for introducing the new family based on congruent results supporting the establishment of a new family.

## Figures and Tables

**Table 1:** DNA sequences and GenBank numbers used for the phylogenetic analyses in this study. The ex-type strains are in bold and the new taxon introduced in this study is indicated in blue.

Taxon	Strain Number	GenBank Accession Numbers		
		*LSU*	*SSU*	*TEF1α*
***Acrocalymma aquatica***	**MFLUCC 11-0208**	JX276952	JX276953	-
***Acrocalymma fici***	**CBS 317.76**	KP170712	-	-
***Acrocalymma medicaginis***	**CPC 24340**	KP170713	-	-
*Acrocalymma medicaginis*	CPC 24341	KP170714	-	-
*Acrocalymma medicaginis*	CPC 24345	KP170718	-	-
***Acrocalymma pterocarpi***	**MFLUCC 17-0926**	MK347949	MK347840	-
*Aigialus grandis*	BCC 20000	GU479775	GU479739	GU479839
*Aigialus mangrovis*	BCC 33563	GU479776	GU479741	GU479840
*Aigialus parvus*	BCC 18403	GU479778	GU479743	GU479842
*Aigialus rhizophorae*	BCC 33572	GU479780	GU479745	GU479844
*Aliquandostipite khaoyaiensis*	CBS 118232	GU301796	-	GU349048
***Amniculicola immersa***	**CBS 123083**	FJ795498	GU456295	GU456273
***Amniculicola lignicola***	**CBS 123094**	EF493861	EF493863	-
***Amniculicola parva***	**CBS 123092**	GU301797	GU296134	GU349065
*Amorosia littoralis*	NN 6654	AM292055	AM292056	-
***Anastomitrabeculia didymospora***	**MFLUCC 16-0412**	MW412978	MW412977	MW411338
***Anastomitrabeculia didymospora***	MFLUCC 16-0417	MW413899	MW413898	MW411339
***Angustimassarina populi***	**MFLUCC 13-0034**	KP888642	KP899128	KR075164
***Angustimassarina quercicola***	**MFLUCC 14-0506**	KP888638	KP899124	KR075169
***Anteaglonium abbreviatum***	**ANM 925a**	GQ221877	-	-
*Anteaglonium globosum*	SMH 5283	GQ221911	-	GQ221919
*Anteaglonium parvulum*	MFLUCC 14-0821	KU922915	KU922916	-
***Antealophiotrema brunneosporum***	**CBS 123095**	LC194340	LC194298	LC194382
*Aquasubmersa japonica*	HHUF 30468	LC061586	LC061581	-
***Aquasubmersa japonica***	**HHUF 30469**	LC061587	LC061582	-
***Aquasubmersa mircensis***	**MFLUCC 11-0401**	JX276955	JX276956	-
*Arthonia dispersa*	UPSC 2583	AY571381	AY571379	-
*Ascocratera manglicola*	BCC 09270	GU479782	GU479747	GU479846
*Ascocylindrica marina*	MD6011	KT252905	KT252907	-
*Ascocylindrica marina*	MD6012	KT252906	-	-
*Ascocylindrica marina*	MF416	MK007123	MK007124	-
*Bahusandhika indica*	GUFCC 18001	KF460274	-	-
***Bambusicola massarinia***	**MFLUCC 11-0389**	JX442037	JX442041	-
*Berkleasmium micronesicum*	BCC 8141	DQ280272	DQ280268	-
*Berkleasmium nigroapicale*	BCC 8220	DQ280273	DQ280269	-
*Bimuria novae-zelandiae*	CBS 107.79	AY016356	AY016338	DQ471087
***Botryosphaeria dothidea***	**CBS 115476**	AY928047	EU673173	AY236898
*Brevicollum hyalosporum*	MAFF 243400	LC271239	LC271236	LC271245
***Brevicollum hyalosporum***	**MFLUCC 17-0071**	MG602200	MG602202	MG739516
*Brevicollum hyalosporum*	PUFNI 17628	MH918671	-	-
*Brevicollum versicolor*	HHUF 30591	LC271240	LC271237	LC271246
***Capnodium salicinum***	**CBS 131.34**	DQ678050	DQ677997	-
*Cladosporium cladosporioides*	CBS 170.54	DQ678057	DQ678004	-
*Clematidis italica*	MFLUCC 15-0084	KU842381	KU842382	-
*Corynespora cassiicola*	CBS 100822	GU301808	GU296144	GU349052
*Corynespora smithii*	CABI 5649b	GU323201	-	GU349018
*Crassiparies quadrisporus*	HHUF 30590	LC271241	LC271238	LC271248
***Crassiparies quadrisporus***	**HHUF 30409**	LC100025	LC100017	-
***Crassiperidium octosporum***	**KT 2144**	LC373108	LC373084	LC373120
*Crassiperidium octosporum*	KT 2894	LC373109	LC373085	LC373121
*Crassiperidium octosporum*	KT 3008	LC373110	LC373086	LC373122
*Crassiperidium octosporum*	KT 3029	LC373111	LC373087	LC373123
*Crassiperidium octosporum*	KT 3046	LC373112	LC373088	LC373124
*Crassiperidium octosporum*	KT 3188	LC373113	LC373089	LC373125
*Crassiperidium octosporum*	KT 3468	LC373114	LC373090	LC373126
*Crassiperidium octosporum*	KT 3604	LC373115	LC373091	LC373127
*Crassiperidium octosporum*	KT 3605	LC373116	LC373092	LC373128
*Crassiperidium octosporum*	MM 9	LC373117	LC373093	LC373129
***Crassiperidium quadrisporum***	**KT 27981**	LC373118	LC373094	LC373130
*Crassiperidium quadrisporum*	KT 27982	LC373119	LC373095	LC373131
***Cryptoclypeus oxysporus***	**HHUF 30507**	LC194345	LC194303	LC194390
***Cryptocoryneum akitaense***	**MAFF 245365**	LC194348	LC194306	LC096136
***Cryptocoryneum japonicum***	**MAFF 245370**	LC194356	LC194314	LC096144
***Cryptocoryneum longicondensatum***	**MAFF 245374**	LC194360	LC194318	LC096148
***Cyclothyriella rubronotata***	**CBS 141486**	KX650544	KX650507	KX650519
*Cyclothyriella rubronotata*	CBS 121892	KX650541	-	KX650516
*Cyclothyriella rubronotata*	CBS 385.39	MH867543	-	-
*Cyclothyriella rubronotata*	CBS 419 85	GU301875	-	GU349002
*Delitschia didyma*	UME 31411	DQ384090	AF242264	-
*Delitschia winteri*	CBS 225.62	DQ678077	DQ678026	DQ677922
*Dendrographa decolorans*	Ertz 5003	AY548815	AY548809	-
*Dendrographa leucophaea f. minor*		AF279382	AF279381	-
*Dendryphion europaeum*	CPC 22943	KJ869203	-	-
***Dendryphion europaeum***	**CPC 23231**	NG_059120	-	-
*Dendryphion nanum*	MFLUCC 16-0975	MG208132	-	MG207983
***Didymosphaeria rubi-ulmifolii***	**MFLUCC 14-0023**	KJ436586	KJ436588	-
*Dissoconium aciculare*	CBS 204.89	GU214419	GU214523	-
*Ernakulamia cochinensis*	PRC 3992	LT964670	-	-
*Flavomyces fulophazii*	CBS 135761	KP184040	KP184082	-
***Fuscostagonospora cytisi***	**MFLUCC 16-0622**	KY770978	KY770977	KY770979
***Fuscostagonospora sasae***	**CBS 139687**	AB807548	AB797258	-
***Fusculina eucalyptorum***	**CBS 145083**	MK047499	-	-
***Gordonomyces mucovaginatus***	**CBS 127273**	JN712552		
*Halojulella avicenniae*	JK 5326A	GU479790	GU479756	-
*Halojulella avicenniae*	BCC 20173	GU371822	GU371830	GU371815
*Halojulella avicenniae*	PUFD542	MK026757	MK026754	-
*Halojulella avicenniae*	BCC 18422	GU371823	GU371831	GU371816
*Halojulella avicenniae*	BCC28357	KC555567	KC555565	-
*Halojulella avicenniae*	GR00584	KC555568	KC555566	-
***Halotthia posidoniae***	**BBH 22481**	GU479786	GU479752	-
***Helminthosporium aquaticum***	**MFLUCC 15-0357**	KU697306	KU697310	-
*Helminthosporium velutinum*	MAFF 243854	AB807530	AB797240	-
*Helminthosporium velutinum*	MFLUCC 13-0243	KU697305	-	-
***Helminthosporium velutinum***	**MFLUCC 15-0423**	KU697304	-	-
***Hermatomyces iriomotensis***	**HHUF 30518**	LC194367	LC194325	LC194394
***Hermatomyces tectonae***	**MFLUCC 14-1140**	KU764695	KU712465	KU872757
***Hermatomyces thailandica***	**MFLUCC 14-1143**	KU764692	KU712468	KU872754
***Hobus wogradensis***	**TI**	KX650546	KX650508	KX650521
***Hysterium angustatum***	**CBS 236.34**	FJ161180	GU397359	FJ161096
*Hysterium angustatum*	MFLUCC 16-0623	MH535893	MH535885	MH535878
***Jahnula seychellensis***	**SS2113**	EF175665	EF175643	-
***Latorua caligans***	**CBS 576.65**	KR873266	-	-
***Latorua grootfonteinensis***	**CBS 369.72**	KR873267	-	-
***Lentimurispora urniformis***	**MFLUCC 18-0497**	MH179144	MH179160	MH188055
***Leptosphaeria doliolum***	**CBS 505.75**	GQ387576	GQ387515	GU349069
***Leptoxyphium cacuminum***	**MFLUCC 10-0049**	JN832602	JN832587	-
***Leucaenicola phraeana***	**MFLUCC 18-0472**	MK348003	MK347892	-
***Lignosphaeria fusispora***	**MFLUCC 11-0377**	KP888646	-	-
***Lignosphaeria thailandica***	**MFLUCC 11-0376**	KP888645	-	-
***Lindgomyces ingoldianus***	**ATCC 200398**	AB521736	AB521719	-
***Longiostiolum tectonae***	**MFLUCC 12 0562**	KU764700	KU712459	-
*Lophiotrema eburnoides*	HHUF 30079	LC001707	LC001706	-
***Lophiotrema nucula***	**CBS 627.86**	GU301837	GU296167	GU349073
***Macrodiplodiopsis desmazieri***	**CBS 140062**	KR873272	-	-
*Magnicamarosporium diospyricola*	MFLUCC 16-0419	KY554212	KY554211	KY554209
*Massarina eburnea*	CBS 473.64	GU301840	GU296170	-
***Massariosphaeria phaeospora***	**CBS 611.86**	GU301843	GU296173	-
*Mauritiana rhizophorae*	BCC 28866	GU371824	GU371832	GU371817
*Medicopsis romeroi*	CBS 122784	EU754208	EU754109	KF015679
***Medicopsis romeroi***	**CBS 252.60**	EU754207	EU754108	KF015678
*Medicopsis romeroi*	CBS 132878	KF015622	KF015648	KF015682
*Murispora rubicunda*	IFRD 2017	FJ795507	GU456308	GU456289
***Neoastrosphaeriella krabiensis***	**MFLUCC 11-0025**	JN846729	JN846739	-
***Neohendersonia kickxii***	**CBS 112403**	KX820266	-	-
*Neohendersonia kickxii*	CBS 122938	KX820268	-	-
*Neohendersonia kickxii*	CBS 114276	KX820267	-	-
*Neohendersonia kickxii*	CPC 24865	KX820270	-	-
*Neohendersonia kickxii*	CBS 122941	KX820269	-	-
***Neomassaria fabacearum***	**MFLUCC 16-1875**	KX524145	KX524147	KX524149
***Neomassaria formosana***	**NTUCC 17-007**	MH714756	MH714759	MH714762
***Neomassarina chromolaenae***	**MFLUCC 17-1480**	MT214466	MT214419	MT235785
***Neomassarina pandanicola***	**MFLUCC 16-0270**	MG298945	-	MG298947
***Neomassarina thailandica***	**MFLUCC 10-0552**	KX672157	KX672160	KX672163
*Neomassarina thailandica*	MFLUCC 17-1432	MT214467	MT214420	MT235786
***Neotorula aquatica***	**MFLUCC 150342**	KU500576	KU500583	-
***Neotorula submersa***	**KUMCC 15-0280**	KX789217	-	-
***Occultibambusa bambusae***	**MFLUCC 13-0855**	KU863112	KU872116	-
***Occultibambusa pustula***	**MFLUCC 11-0502**	KU863115	KU872118	-
*Ohleria modesta*	MGC	KX650562	-	KX650533
***Ohleria modesta***	**CBS 141480**	KX650563	KX650513	KX650534
*Paradictyoarthrinium diffractum*	MFLUCC 13-0466	KP744498	KP753960	-
*Paradictyoarthrinium diffractum*	MFLUCC 12-0557	KP744497	-	-
*Paradictyoarthrinium hydei*	MFLUCC 13-0465	MG747497	-	-
*Paradictyoarthrinium tectonicola*	MFLUCC 13-0465	KP744500	KP753961	-
***Paradictyoarthrinium tectonicola***	**MFLUCC 12-0556**	KP744499	-	-
***Periconia thailandica***	**MFLUCC 17-0065**	KY753888	KY753889	-
***Phaeoseptum aquaticum***	**CBS 123113**	JN644072	-	-
*Phaeoseptum terricola*	MFLUCC 10-0102	MH105779	MH105780	MH105781
*Phyllosticta capitalensis*	CBS 226.77	KF206289	KF766300	-
***Piedraia hortae***	**CBS 480.64**	GU214466	-	-
***Polyplosphaeria fusca***	**CBS 125425**	AB524607	AB524466	AB524822
***Preussia lignicola***	**CBS 363.69**	DQ384098	DQ384087	-
*Preussia lignicola*	CBS 264.69	GU301872	GU296197	GU349027
***Pseudoastrosphaeriella bambusae***	**MFLUCC 11-0205**	KT955475	KT955455	KT955437
***Pseudoastrosphaeriella longicolla***	**MFLUCC 11-0171**	KT955476	KT955456	KT955438
***Pseudoastrosphaeriella thailandensis***	**MFLUCC 10-0553**	KT955477	KT955456	KT955439
***Pseudolophiotrema elymicola***	**HHUF 28984**	LC194381	LC194339	LC194418
***Pseudomassariosphaeria bromicola***	**MFLUCC 15-0031**	KT305994	KT305996	KT305999
***Pseudotetraploa curviappendiculata***	**CBS 125426**	AB524610	AB524469	AB524825
*Quadricrura septentrionalis*	CBS 125428	AB524617	AB524476	AB524832
*Racodium rupestre*	L346	EU048583	EU048575	-
*Racodium rupestre*	L424	EU048582	EU048577	-
***Ramusculicola thailandica***	**MFLUCC 13-0284**	KP888647	KP899131	KR075167
*Rimora mangrovei*	JK 5246A	GU301868	GU296193	
*Roccella fuciformis*	Tehler 8171	FJ638979	-	-
***Rostriconidium aquaticum***	**KUMCC 15-0297**	MG208144	-	MG207995
*Rostriconidium aquaticum*	MFLUCC 16-1113	MG208143	-	MG207994
*Salsuginea ramicola*	KT 2597.1	GU479800	GU479767	GU479861
*Salsuginea ramicola*	CBS 125781	MH877872	-	-
*Scorias spongiosa*	CBS 325.33	MH866910	GU214696	-
*Seriascoma didymospora*	MFLUCC 11-0179	KU863116	KU872119	-
*Sigarispora arundinis*	JCM 13550	AB618998	AB618679	LC001737
***Sigarispora ravennica***	**MFLUCC 14-0005**	KP698414	KP698415	-
*Splanchnonema platani*	CBS 222.37	KR909316	KR909318	KR909319
*Sporidesmioides thailandica*	KUMCC 16-0012	KX437758	KX437760	KX437767
***Sporidesmioides thailandica***	**MFLUCC 13-0840**	NG_059703	NG_061242	KX437766
*Sporormia fimetaria*	UPS:Dissing Gr.81.194	GQ203729	-	-
***Sporormiella minima***	**CBS 52450**	DQ468046	-	DQ468003
***Stagonospora pseudocaricis***	**CBS 135132**	KF251762	KF251259	KF252741
***Stemphylium vesicarium***	**CBS 191.86**	DQ247804	DQ247812	DQ471090
*Stemphylium vesicarium*	CBS 714.68	DQ678049	DQ767648	DQ677888
***Sulcatispora acerina***	**KT2982**	LC014610	LC014605	LC014615
***Sulcosporium thailandicum***	**MFLUCC 12-0004**	KT426563	KT426564	-
***Teichospora quercus***	**CBS 143396**	MH107966	-	MH108030
***Tetraplosphaeria sasicola***	**KT 563**	AB524631	AB524490	AB524838
***Torula gaodangensis***	**MFLUCC 17-0234**	NG_059827	NG_063641	-
*Torula herbarum*	CBS 111855	KF443386	KF443391	KF443403
***Triplosphaeria maxima***	**MAFF 239682**	AB524637	AB524496	-
***Tubeufia chiangmaiensis***	**MFLUCC 11-0514**	KF301538	KF301543	KF301557
***Tubeufia javanica***	**MFLUCC 12-0545**	KJ880036	KJ880035	KJ880037
*Vargamyces aquaticus*	CBS 639.63	KY853539	-	-
*Vargamyces aquaticus*	HKUCC 10830	DQ408575	-	-
***Versicolorisporium triseptatum***	**HHUF 28815**	AB330081	AB524501	-
*Westerdykella dispersa*	CBS 297.56	MH869191	-	-
***Westerdykella ornata***	**CBS 379.55**	GU301880	GU296208	GU349021
***Xenomassariosphaeria rosae***	**MFLUCC 15-0179**	MG829092	MG829192	-

**Table 2:** Divergence time estimates obtained from BEAST analysis. The median and the 95% Highest Posterior Density are provided in million years ago (MYA). The geological time scales are given based on the median node age.

Nodes	Node Age	Geological Time Period
*Arthoniomycetes–Dothideomycetes*	323 (310–349)	Carboniferous
*Dothideomycetes* crown group	263 (216–313)	Permian
*Hysteriales*–*Pleosporales*	236 (188–300)	Triassic
*Pleosporales* crown group	206 (171–254)	Triassic
*Capnodiales* crown group	147 (99–200)	Jurassic
*Anastomitrabeculiaceae* stem group	84 (52–116)	Cretaceous
*Aigialaceae–Aigialus* sp.	37 (18–56)	Eocene
*Anastomitrabeculiaceae* crown group	2.6 (0.19–6.61)	Neogene

**Table 3:** Divergence time estimates obtained from BEAST analysis for families with similar morphology to *Anastomitrabeculiaceae*. The crown age and the stem age are provided in million years ago (MYA).

Families	Crown Age	Stem Age
*Aigialaceae*	102	141
*Amniculicolaceae*	90	177
*Anastomitrabeculiaceae*	2.6	84
*Anteagloniaceae*	52	98
*Bambusicolaceae*	29	57
*Cyclothyriellaceae*	66	95
*Delitschiaceae*	78	131
*Didymosphaeriaceae*	47	81
*Fuscostagonosporaceae*	26	63
*Lindgomycetaceae*	31	92
*Neomassariaceae*	82	131
*Pseudoastrosphaeriellaceae*	56	147
*Tetraplosphaeriaceae*	91	189

## Data Availability

All sequences generated in this study were submitted to GenBank.
